# Fasudil attenuates lipopolysaccharide-induced cognitive impairment in C57BL/6 mice through anti-oxidative and anti-inflammatory effects: Possible role of aquaporin-4

**DOI:** 10.1016/j.ibneur.2024.10.004

**Published:** 2024-10-24

**Authors:** Sahra Jalalkamali, Mohsen Ghahremani, Vida Jashn, Negin Sadat Lajevardi, Sevda Mahdipoor Koloor, Seyede Zohreh Jazaeri, Javad Fahanik-babaei

**Affiliations:** a Electrophysiology Research Center, Neuroscience Institute, Tehran University of Medical Sciences, Tehran, Iran; b Department of Neuroscience, Faculty of Advanced Technologies in Medicine, Iran University of Medical Sciences, Tehran, Iran; c Division of Neuroscience, Cellular and Molecular Research Center, Iran University of Medical Sciences, Tehran, Iran

**Keywords:** Fasudil, Lipopolysaccharide, Inflammation, Oxidative Stress, Cognitive Dysfunctions, AQP-4

## Abstract

**Introduction:**

Processes that generate systemic inflammation are strongly associated with neurodegenerative diseases. This study aimed to explore the potential anti-oxidative and anti-inflammatory effects of fasudil and its role in modulating aquaporin-4 (AQP-4) to improve cognitive impairment in a systemic inflammation model induced by lipopolysaccharide (LPS).

**Method:**

fourty C57BL/6 mice were assigned to four groups, including sham, LPS, sham+fasudil, and LPS+fasudil). Intraperitoneal LPS was given (500 μg/kg/day) at hours 0, 24, 48, and 72, and fasudil (30 mg/kg) administered intraperitoneal injections 2 hours after LPS injection. The open field, Y-maze, and Novel object tasks was used to assess learning and memory. The levels of malondialdehyde (MDA), superoxide dismutase (SOD), interleukin-10 (IL-10), and tumor necrosis factor-α (TNF-α) in the hippocampus also measured as markers of oxidative stress and inflammation. Furthermore, the expression of AQP-4 measured in the intact and experimental groups.

**Results:**

The results showed that Fasudil significantly improved memory and anxiety behavior induced by LPS in the open field maze, spatial recognition memory in the Y-maze, and performance in the novel object recognition task. It also mitigates hippocampal MDA and SOD levels. Additionally, fasudil ameliorated LPS-induced hippocampal levels of TNFα and IL-10 and increased hippocampal levels of AQP-4 expression in mice.

**Conclusion:**

Our results suggest that fasudil in the LPS model of systemic inflammation could improve cognition by suppressing oxidative stress and inflammation and increasing AQP-4 protein expression. These findings highlighted the potential of fasudil as a neuroprotective agent. However, further research is required to fully understand its neuroprotective properties in the treatment of neurodegenerative disorders.

## Highlights

• Structure–function of brain is severely damaged in systemic inflammation model.
• systemic inflammation causes neurobehavioral problems, cognition and memory impairment in mice model.
• By increasing AQP-4 levels, fasudil helps restore brain water homeostasis and mitigates the inflammatory response.

## Graphical Abstract

**Figure:**
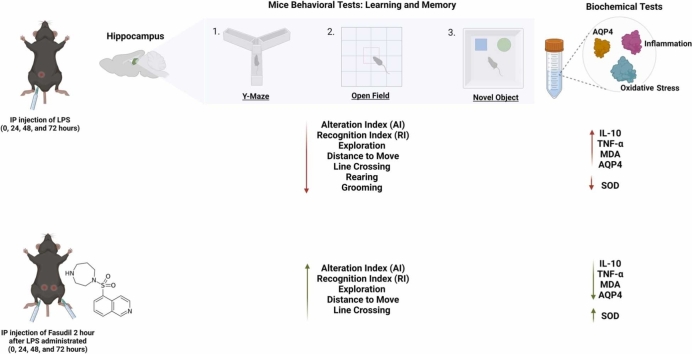

## 1 Introduction

Systemic inflammation induced by various processes, such as extensive burns, sepsis, pancreatitis, major surgeries, and some chronic inflammatory conditions such as autoimmune diseases, diabetes mellitus, obesity, cancer, and aging, have been found to have a strong relationship with neurological diseases. Inflammation can lead to neurodegeneration and acute behavioral and cognitive changes (Millán Solano et al., 2023, Sankowski and Mader, 2015). The association between systemic inflammation and cognitive deficits is well documented, with systemic inflammation being established as a potential mechanism underlying cognitive decline (Lin et al., 2018). In contrast, cognitive impairment can result in various motor and sensory impairments, including muscle weakness, poor motor coordination (Orsini et al., 2018), and hearing or vision loss. These impairments can limit the neural resources required for optimal cognitive tasks and may lead to depression, social isolation, and a lack of physical activity, further contributing to cognitive impairment (Whitson et al., 2018).

Intraperitoneal injection of lipopolysaccharide (LPS) is a well-established method for inducing systemic inflammation and studying its effects on brain and neurological diseases. Currently, this method is used to investigate the role of inflammation in neurodegenerative diseases such as Parkinson's disease (PD) and Alzheimer's disease (AD), and its potential mechanisms (Qin et al., 2007). Moreover, systemic and central administration of LPS has been shown to cause cognitive impairment, elevated levels of pro-inflammatory cytokines, and neuroinflammation, which can contribute to neurodegenerative processes (Zhao et al., 2019). Evidence suggests that inflammation in various peripheral organs can trigger inflammation in the brain and contribute to several neurological and psychiatric disorders (Sun et al., 2022). The detailed mechanism underlying cognitive decline due to systemic inflammation remains unclear, however, data from animal models suggest various possible mechanisms involving chronic inflammation (Allison and Ditor, 2014).

Aquaporin-4 (AQP-4), an essential part of the glymphatic system, is the most abundantly expressed aquaporin in the Central Nervous System (CNS) and has a vital role in both edema regulation and different cognitive performances (Fukuda and Badaut, 2012, Wang et al., 2022). These protein channels are mainly located in astrocytes and facilitate water movement across the astrocytic membrane. Astrocytes contribute to the maintenance of the appropriate balance between ions and water in the brain, which is essential for normal neuronal function, by controlling water homeostasis in the Extracellular Space (ECS) (Papadopoulos and Verkman, 2013, Szu and Binder, 2022). Therefore, AQP-4 may be essential for cognitive impairment following systemic inflammation, which has not been investigated until now. On the other hand, there is no effective therapy to treat cognitive sequelae associated with systemic infectious diseases, but anti-inflammatory and antioxidant therapies may be potential therapeutic targets (Reis and Castro-Faria-Neto, 2022).

Fasudil, a Rho kinase inhibitor, exerts its effects through various mechanisms, including modulation of inflammation and oxidative stress (Liu et al., 2022) and improving motor and cognitive functions. Studies have shown that peripheral administration of fasudil can reverse motor and cognitive deficits in mouse models of Alzheimer's disease (AD) and Parkinson’s disease (PD) (Killick et al., 2023, Lopez-Lopez et al., 2020). One study demonstrated that pre-treatment of mice with fasudil before exposure to lung-damaging LPS resulted in an upregulation of AQP-5 mRNA and protein levels, which were decreased by exposure to LPS. Furthermore, fasudil pretreatment led to downregulation of IL-6 in the lungs, peripheral blood, and bronchoalveolar fluid (BALF) of mice exposed to LPS (Wang et al., 2019). These findings suggest that fasudil may improve motor and cognitive dysfunctions in an LPS model of C57BL/6 mice by modulating the expression of AQP-4, which is essential for edema formation or elimination, as well as cognitive and motor functions, particularly in neurological diseases (Jazaeri et al., 2023).

Given the significant role of neuroinflammation in neurodegenerative diseases, our study aimed to investigate the effects of Fasudil on motor and cognitive functions, and its anti-inflammatory and antioxidant effects on LPS-induced neuroinflammation in C57BL/6 mice. Additionally, we examined the effects of fasudil on the expression of AQP-4, which is crucial for cognitive impairment caused by systemic inflammation.

## 2 Material and method

### 2.1 Design of experiment

**Fig. 1:**
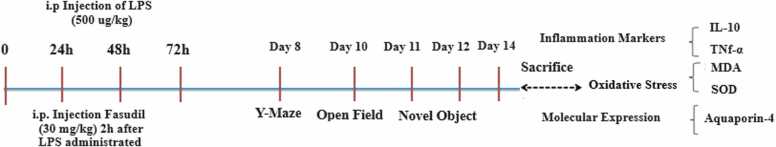
Time line of the study's experimental design.

40 male mice C57BL/6 (30–35 g) were assigned to 4 experimental groups, including sham, LPS, sham+ fasudil (30 mg/kg), and fasudil-treated LPS. Intraperitoneal LPS was administered (500 μg/kg/day) at 0, 24, 48, and 72 h. In the sham+fasudil and fasudil-treated LPS groups, fasudil was administered intraperitoneal injections 2 hours after LPS injection for 4 days. Mice in the sham group were administered normal saline. All groups were evaluated from the 8th to 12th day after the first LPS injection using behavioral tests. On the 14th day, brain samples were collected from the animals for biochemical and molecular experiments. Mortality caused by LPS administration was at least one animal in each group. All experiments were performed between 9 a.m. and 4 p.m. NIH guidelines for the care and use of laboratory animals (NIH No. 8023, revised 1978) were followed for all the experimental protocols and procedures. This study was approved by the Ethics and Research Committee of the Tehran University of Medical Sciences (IR TUMS.NI.REC.1400,05). Parameters measured and the time intervals are presented in a scheme of experimental diagram in Fig. 1.

### 2.2 Behavioral studies

#### 2.2.1 Open-Field Test (OFT)

In light of the short-term and long-term effects of lipopolysaccharide (LPS), behavioral assessments commenced eight days following the initial LPS injection in the inflammatory animal model. This timing was chosen to account for the dynamics of the immune response, the stabilization of the model, and the associated behavioral changes. The Open Field Test (OFT) is a common technique used to evaluate rodents' general activity and exploratory behavior, measuring both the quantity and quality of activity (Lopez-Lopez et al., 2020). The OFT was conducted on the animals for six minutes in a plexiglass box (measuring 30 cm in length, 30 cm in width, and 15 cm in height), divided into nine equal squares. The progression of arm movements was tracked automatically by a computerized system (EthoVision, Noldus, Version 11) using a Charge-Coupled Device (CCD) camera located 200 cm above the maze's center. The mice's exploratory activities were determined by the number of times they stood on their hindlimbs, while their locomotor activity was assessed by the number of segments they crossed with their four paws. Other behaviors analyzed included distance traveled and grooming.

#### 2.2.2 Y-maze

The Y-maze's spontaneous alternation test is widely used to assess mice's short-term spatial memory. This test is based on rodents' natural curiosity to explore new places, and is conducted without any positive or negative stimuli in the maze. This can be evaluated by permitting the animal to explore all three arms of the maze (Wang et al., 2019). Key brain regions perform the task, including the hippocampus, prefrontal cortex, septum, and basal forebrain. A mouse with intact working memory and, consequently, healthy prefrontal cortex functioning will recall the arms it has previously visited and tend to enter a less often visited arm (Wang et al., 2019). The trial was executed in plexiglass equipment composed of 3 arms (50 cm in length, 10 cm in breadth, and 10 cm in height) (Kim et al., 2023). The arms were angled 120° to each other. Before testing the spontaneous alternations, animals' habituation was performed for 30 sec and then returned to their home cage. Mice were located in the middle of the equipment and could move freely through the Y-maze for six minutes (without using water or food reinforcers). The progression of arm movements was tracked automatically by a computerized system (EthoVision, Noldus, Version 11) using a Charge-Coupled Device (CCD) camera located 280 cm above the maze's center. All four paws must be inside the arm for valid entry. After six min, the animals were returned to their home cage. Correct alternations were counted if the animal entered the three arms successively. The percentage of correct alternations was evaluated using the following equation:

(Number of alterations/total arm entries-2) ×100.

#### 2.2.3 Novel Object

The Novel Object Recognition (NOR) test is a memory assessment task used to evaluate cognition and recognition memory in rodent models of CNS disorders. This test is based on rodents' natural curiosity to explore a novel object more than the familiar object. The test consists of three sessions: habituation, familiarization, and test sessions. For habituation, the animals were allowed to explore an empty arena on the first day. An open-field square box (50 cm in length, 50 cm in breadth, and 50 cm in height) was used for 5 minutes. One day after habituation, during the familiarization session, each animal was placed in an arena with two similar objects attached to the floor at an equal distance from the walls, and each other mice investigated two familiar subjects. The animals were taken and returned to their cages after 10 minutes. Twenty-four hours following familiarization, the mice were placed in the arena for 10 min with a novel object replaced by one of the familiar objects. The new object was the same material, height, and volume as the known object but was different in color and form. Item exploration was defined as the animal's nostrils in the zone at least 2 cm from the item. The animals had never seen our goods before they were examined. After each trial, the box was carefully cleaned with 70 % ethanol to avoid any possible influence of olfactory stimuli. The total time spent investigating each object was automatically recorded using a video camera-based system (EthoVision, Noldus, Version 11). A comparison of total exploration between familiar and novel objects was assayed, and the Recognition Index (RI) was computed using the following formula: [RI = (TN) / (TN + TF) × 100]. Where TN is the novel object spent time, TF is the familiar object spent time.

### 2.3 Biochemical studies

#### 2.3.1 Sample preparation

After the behavioral tests, the mice were sacrificed under ketamine anesthesia (150 mg/kg). The brain was immediately removed, and the hippocampus was isolated, cleaned, washed with ice-cold Phosphate-Buffered Saline (PBS), and homogenized in cold RIPA buffer (pH 7.4). This assay was conducted after centrifuging the supernatant (1,0000 g, 4 °C, 10 min). Duplicate measurements were performed for all parameters. The total protein concentration was determined using the Bradford method (Bradford, 1976).

#### 2.3.2 Oxidative Stress

Reactive Oxygen Species (ROS) are naturally produced in the body and play a crucial role in oxidative stress, leading to cellular damage and contributing to various diseases. Two important antioxidant defense mechanisms against oxidative stress are superoxide dismutase (SOD) and malondialdehyde (MDA) (Kim et al., 2023). Superoxide dismutase (SOD) activity was determined using a specific assay kit (Kiazist, Live Science, Iran). Briefly, the supernatant was incubated with xanthine and xanthine oxidase in potassium phosphate buffer for 30 minutes, and Nitro Blue Tetrazolium (NBT) was added. In the superoxide dismutase (SOD) assay, the generation of blue formazan serves as a crucial marker for the production of superoxide anions (O2·−). This reaction entails the reduction of nitroblue tetrazolium (NBT), a yellow water-soluble compound, by superoxide radicals that are produced during oxidative stress. Awavelength of 570 nm was used to monitor the formation of blue formazan (n = 5 per group). The extent of lipid oxidation was determined by measuring the MDA levels, in which MDA reacts with thiobarbituric acid (TBA) to form a colored complex at a maximum absorbance of 535 nm. MDA is a low-weight lipid peroxidation product that originates from the decomposition of highly reactive lipid hydroperoxides [21 (Spickett et al., 2010). We used the measuring kit (Kiazist, LiveScience, Iran) factory prototype to measure it.

#### 2.3.3 ELISA to measure inflammatory factors

To assess inflammatory factors, the amount of each cytokine, TNF-α and IL-10, also known as pro- and anti-inflammatory factors, was measured using the appropriate kits and antibodies using the ELISA method (Karmania pars gene, Iran). In summary, the ELISA plate was filled with 50 μL of the samples and standards and incubated for an hour. After adding the HRP-AVIDIN and the detection antibody, the plates were cleaned using the washing buffer. The plates were washed, and the substrate was added after an hour of incubation. The stopping solution halted the reaction after 15 min, and an ELISA reader (Karen Teb, Iran) was used to measure the Optical Density (OD) at 450 nm.

### 2.4 Western blotting to measure AQP4 expression

Western blot analyses were performed as previously reported, with a few modifications (Nazari et al., 2022). RIPA buffer was used to lyse the tissue to perform western blotting. Centrifugation was used to extract the lysates at 14,000 rpm for 20 minutes at 4°C. The Bradford Protein Quantification kit (DB0017, DNAbioTech, Iran) measured the protein concentration following the manufacturer's instructions. 2X Laemmli sample buffer was added in an equal volume to the tissue lysates. After boiling for five minutes, lysates (20 μg) were subjected to SDS-PAGE and then placed on a 0.2 μm immune-BlotTM Polyvinylidene Difluoride (PVDF) membrane (Cat No: 162–017777; Bio-Rad Laboratories, CA, USA). The membranes were blocked for one hour at room temperature using a TBS solution that contained 1 % (v/v) Tween 20 and 5 % (w/v) BSA (Cat No. A-7888; Sigma Aldrich, MO, USA). The membranes were incubated for one hour at room temperature with anti-β actin-loading control antibodies (Cat No: ab8227, Abcam) and anti-Aquaporin 4 (Cat No: ab259318, abcam) in solution that contained TBS + 0,1 % Tween 20 + 3 % BSA. Following three TBS + 0.1 % Tween 20 washes, The membranes were n incubated with goat anti-rabbit IgG H&L (HRP) (Cat No: ab6721; Abcam) secondary antibody after being cleaned three times with TBST. The membranes were incubated for one to two minutes, in accordance with the manufacturer's instructions, to an ECL kit for chemiluminescence detection, and the immuno-reactive bands were visualized using the Amersham ECL prime western blotting detection (GE Healthcare Life Sciences). The expression of proteins was compared to β-actin. Protein band densitometry was carried out using the gel analyzer Version 2010a software (NIH, USA), and the calculated values were compared between groups. Specifically, the percentage area under the curve of each band was divided by the percentage area under the curve of its corresponding actin band.

### 2.5 Statistical analysis

All measurements were performed by an independent investigator blinded to the experimental conditions. The data were analyzed, and graphs were drawn using Graph Pad Prism software 8.0. Behavioral tests in groups were conducted using one-way ANOVA. We also used One-way ANOVA with Tukey’s post hoc to evaluate and compare biochemical and histological tests in groups and two pulses in groups. Additionally, P<0.05 was considered significant, and if significant, Tukey's post hoc test was used. Data are expressed as Mean ± SEM.

## 3 Results

### 3.1 Behavioral results

#### 3.1.1 Open-Field Test (OFT)

**Fig. 2:**
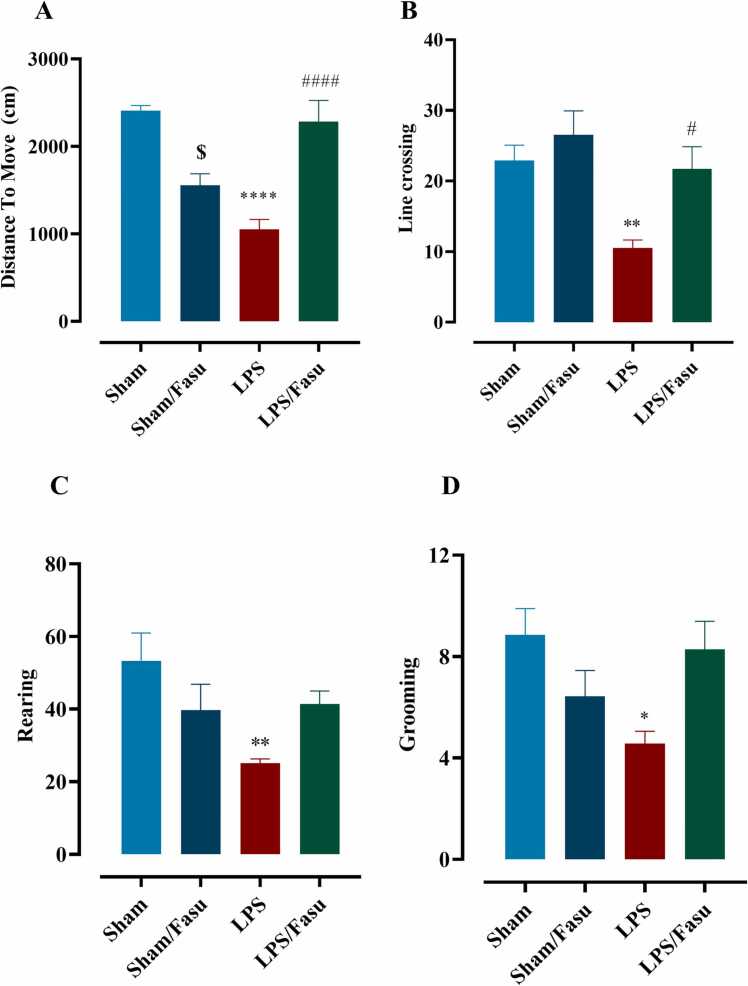
**Behavioral testing of open field in LPS and post-treatment with fasudil**. Ethovision software tracking uses the contrast of black mice on a white background in an open field box to determine the center point to measure multiple open field parameters. Diagram depicted the time spent at different locations within the open field box over 6 minutes in mice of sham, sham+ fasudil, LPS, and LPS+Fasudil groups for (A) Distance to move and (B) The numbers of line crossings in the central and the numbers of (C) Rearing and (D) Grooming as anxiety behavior in the open field test. (*P<0.05, **P<0.01, ****P < 0.0001 sham compared to LPS; $P<0.05 sham compared to Sham+Fasu; #P<0.05, ####P<0.0001 for LPS compared to LPS+fasudil (n= 8±1)). All graphs were plotted as mean± SEM.

The results of mobility data were performed to evaluate the extent of stress-related behaviors and the effect of fasudil on improving movement disorder LPS-induced. The results of the present study showed a significant decrease in the distance to move (mean distance moved) and vertical activity (cross count) in group animals of LPS compared with the sham group (F(3, 24)= 21.4; P < 0.0001, for distance to move, F (3, 35)= 7.2; P < 0.0007, for vertical activity, Fig. 2A&B). One-way ANOVA with Tukey’s post hoc analyses indicated that, compared to the sham group, there was a significant effect of LPS on the decrease of distance to move and vertical activity in an open field maze (P<0.0001 and P< 0.05, respectively). Our results also showed that compared to the LPS group, there was a statistically significant increase of LPS + Fasudil in both distances to move and vertical activity (P<0.0001). Our result also indicated that post-treatment affected the number of rearing and grooming behaviors between experiment groups (F(3, 24)= 21.4; P < 0.0001, for rearing, F(3, 35)= 7.2; P < 0.0007, for grooming, Fig. 2A&B). Rearing analysis was performed in the open field test to evaluate the curiosity of mice in the four groups to stand on their hind legs. The results showed a significant decrease in the rearing and grooming count in group animals of LPS compared with the sham group (F (3, 24)= 4.3; P < 0.01, for distance to move, F(3, 24)= 4.2; P < 0.01, for vertical activity, Fig. 2C&D). One-way ANOVA with Tukey’s post hoc analyses indicated a significant reduction in the rearing number in the LPS groups compared with the sham group (P < 0.01); however, there was no considerable effect LPS compared to the LPS+ fasudil group in the rearing number (P = 0.19). The results showed a significant reduction in the number of grooming in the LPS group compared with the sham group (P<0.5). Evaluation of grooming numbers showed a significant increase in the LPS+fasudil groups compared with the LPS group (P<0.05).

#### 3.1.2 Y-Maze

**Fig. 3:**
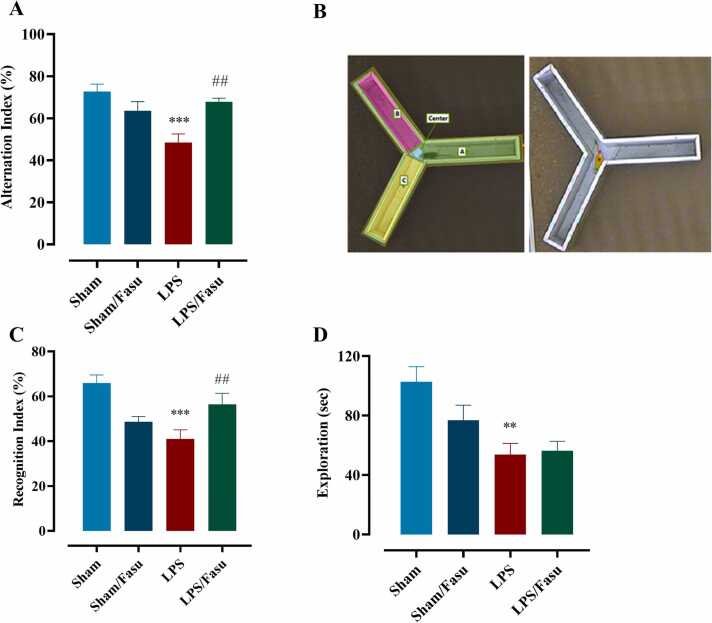
**Animal detection and behavioral measurement of cognitive performance**. The effect of LPS and post-treatment with fasudil on spontaneous alternation in Y-maze test. (A) Y-maze apparatus. (B) The percentage of correct alternations was significantly different between experimental groups. The effect of LPS and post-treatment with fasudil on recognition index and exploration time in novel object recognition test. Comparison of the recognition index assay (C) and total exploration time for familiar and novel objection (D) between experimental groups (**P <0.01 and ***P < 0.001, sham compared to LPS; ##P <0.01 for LPS compared to LPS+fasudil (n= 8±1)). All graphs were plotted as mean± SEM.

The Y-maze test evaluated LPS effects and post-treatment with Fasudil on spatial working memory via spontaneous behavioral alteration. Our result indicated that treatment affected the alteration index between experiment groups (F (3, 36) = 8.72; P< 0.0002). One-way ANOVA with Tukey’s post hoc analyses indicated that, compared to the sham group, there was a significant effect of LPS on Spontaneous Alternation Percentage (SAP) (71.7 ±5.6 vs. 48.4 ± 5.5; P<0.0001). Similar results were obtained from the comparison of the sham + fasudil and LPS groups (63.5±5.8 vs. 48.4±5.5; P<0.05), as well as our results showed that the impairment was mitigated significantly in the LPS + fasudil group compared to the LPS group (67.8±5.6 vs. 48.4±5.5; P < 0.001, Fig. 3A). The results indicated that there was significant cognitive impairment in the LPS group compared with the sham group, and fasudil could improve working memory in LPS-induced animals.

#### 3.1.3 Novel object

Performance on the NOR task was measured by the total exploration time and recognition index to evaluate LPS effects and post-treatment of fasudil. NOR Results indicated that total exploration (Fig. 3C) and recognition index (Fig. 3D) significantly differed among experimental groups. According to our result, the total exploration time for both objects revealed a significant influence between groups [F (1.97, 13.83) = 5.69, P<0.01]. One-way ANOVA with Tukey’s post hoc analyses indicated that there was a significant decrease between sham group animals compared to animals of LPS (P < 0.01); however, there was no significant effect LPS compared to LPS+ fasudil group (P = 0.99). Results of the recognition index also were significantly different among experimental groups [F (2, 23)= 8.11, P<0.0007). One-way ANOVA with Tukey’s post hoc analyses indicated that as compared to the sham, the recognition index was significantly lower in the LPS group (65.91 ± 5.2 % vs. 48.01 ± 5.8 %; P<0.01) and administrate of fasudil could significantly increase the index in LPS+ fasudil group compared to LPS group (56.45 ± 5.6 % vs 48.01 ± 5.8 %; P < 0.01). These results indicate that LPS leads to persistent impairment in recognition memory in mice, and fasudil administration had a significant effect on LPS-induced memory impairment.

### 3.2 Biochemical studies

#### 3.2.1 The effect of antioxidative fasudil on mice administrated LPS

**Fig. 4:**
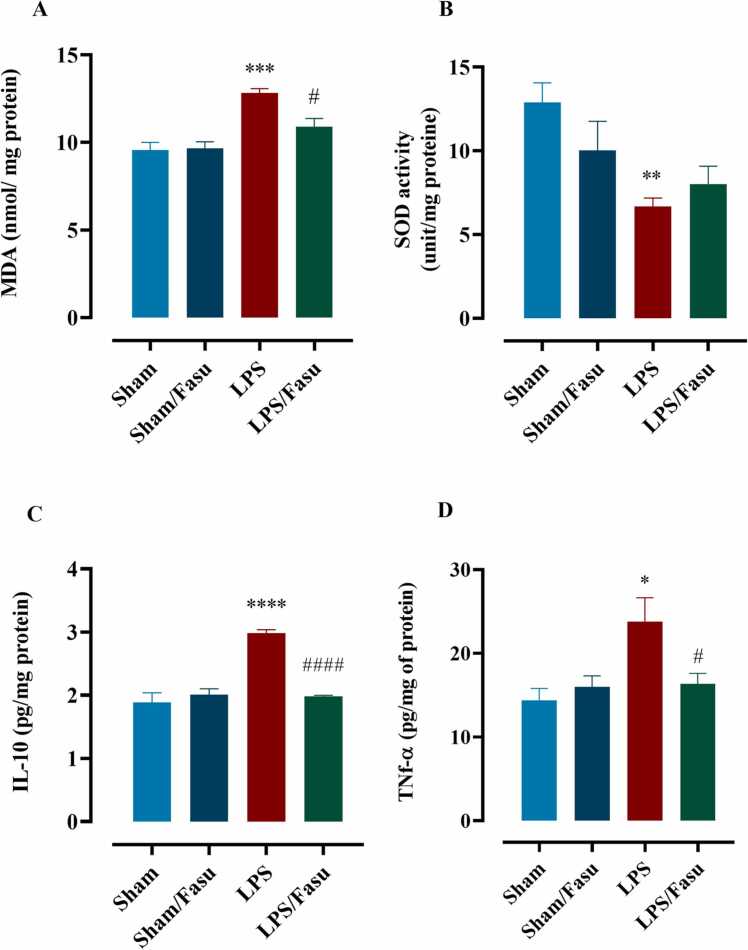
The effect of LPS and post-treatment with fasudil on oxidative stress and inflammation markers. Comparison of the MDA level (A) and SOD activity (D) as oxidative stress markers and levels of IL-10 (C) and TNF-α (D) as markers of inflammatory cytokines between experimental groups. (*P <0.05, **P <0.01, ***P <0.001, ****P < 0.0001 sham compared to LPS; #P <0.05, ####P <0.0001 for LPS compared to LPS+fasudil (n= 5)). All graphs were plotted as mean± SEM.

In addition to behavioral studies, two markers, MDA and SOD, were used to measure fasudil antioxidative effects. Evaluation of brain tissue MDA indicated that there was a significant difference among experimental groups (F (3,16)= 14.9; P <0.0001 Fig. 4A). One-way ANOVA with Tukey’s post hoc analyses indicated that LPS significantly increased MDA level in mice hippocampus structures compared to a sham group (P <0.001). Interestingly, fasudil reduced MDA levels in animals of the LPS+fasudil group compared to the LPS (P <0.05). An assay of SOD activity in hippocampus tissue indicated that there was significantly reduced activity of enzyme among experimental groups (F (3,15)= 5.2; P <0.05Fig. 4B). One-way ANOVA with Tukey’s post hoc test confirmed that the LPS led to a decrease in SOD activity compared to the group sham (P <0.01), and despite, fasudil increased enzyme activity in LPS + fasudil group compared to the LPS group, but still, the increase does not statistically significant (P =0.84).

#### 3.2.2 The effect of fasudil on inflammation markers in mice adminsitrated to LPS

To investigate the effects of fasudil on neuroinflammation induced by LPS, we detected the expression levels of TNF-α and IL-10 in the hippocampus using Elisa. Analysis of IL-10 and TNF-α levels in hippocampal tissue revealed significant differences between experimental groups under study (F (3,12): 30.1; P <0.0001 for IL-10 and F (3,16): 5.2; P <0.01 for TNF-α). One-way ANOVA with Tukey’s post hoc analyses indicated that compared with the sham group, mice in the LPS group possessed higher contents of IL-10 (P<0.0001, Fig. 4C) and TNF-α (P < 0.05; Fig. 4D) in the hippocampus. Interestingly, in the fasudil+LPS group, compared with the LPS group, the levels of IL-10 (P <0.0001) and TNF-α (P <0.05) were significantly reduced after post-treatment with fasudil. In summary, fasudil alleviated neuroinflammation in the hippocampus by reducing the contents of IL-6 and TNF-α in mice that receive LPS.

### 3.3 Western bloting

#### 3.3.1 The effect of fasudil on AQP-4 expression in mice adminsitrated to LPS

**Fig. 5:**
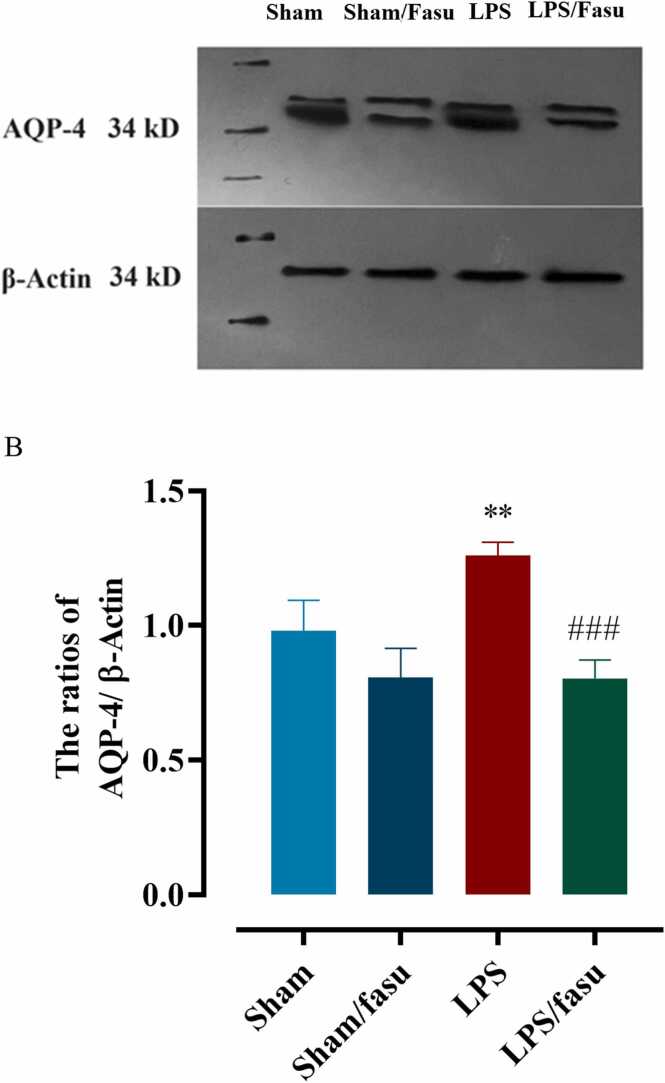
**The effect of LPS and post-treatment with fasudil on the expression of AQP-4 in hippocampus tissue.** (A) Western blot assay for expression of AQP-4 in the tissue of sham and experimental groups. (B) The relative expression levels of AQP-4 protein in mice hippocampus tissue. The relative expression level of AQP-4 protein was normalized against β-actin and presented as the ratio of the values of the experimental group to the normal sham group. (**P <0.01, sham compared to LPS; ###P <0.001 for LPS compared to LPS+fasudil (n= 4)). All graphs were plotted as mean± SEM.

To investigate the effects of fasudil on AQP-4 expression induced by LPS, we detected the expression levels of AQP-4 in the hippocampus using Western blotting (Fig. 4A&B). Analysis of expression levels of AQP-4 in hippocampal tissue revealed significant differences between experimental groups (F (3,12): 23.4; P <0.0001; Fig. 5A). One-way ANOVA with Tukey’s post hoc analyses indicated that compared to the sham group, mice in the LPS group had higher expression of AQP-4 (P<0.01) in the hippocampus tissue. However, in the LPS+fasudil group, as compared to the LPS group, the expression levels of AQP-4 (Figure, p < 0.001) were significantly reduced after fasudil post-treatment (P <0.001 Fig. 5B).

## 4 Discussion

Our study is the first to demonstrate that Fasudil can mitigate LPS-induced behavioral impairments (motor and cognitive) by inhibiting oxidative stress, inflammatory factors, and AQP-4 regulation in the brains of C57BL/6 mice. While previous research has shown the beneficial effects of Fasudil on inflammatory diseases such as lung injury (Wang et al., 2019) and focal cerebral ischemia (Sheikh et al., 2022), our study is unique in its focus on the functional improvements conferred by Fasudil in a model of systemic inflammation, particularly its effects on AQP-4. Consequently, this finding underscores the potential of Fasudil as an inhibitor of Rho-associated protein kinase (ROCK), which is implicated in various cellular processes, including inflammation and apoptosis, such as neurological and neurodegenerative diseases. It highlights the need for further research in this area.

Systemic inflammation, which occurs in many neurological diseases, has been suggested as a risk factor for behavioral dysfunctions, specifically cognitive decline, and anti-inflammatory therapy has been explored as a potential treatment (Leonardo and Fregni, 2023). Fasudil, which may exert its effect by edema modulation, has been shown to have various effects on behavior and cognitive function. Our findings reveal that fasudil positively impacts both motor function, as indicated by increased distance traveled in the open field test, and cognitive function, encompassing spatial memory, recognition memory, and working memory. These cognitive improvements were assessed through various cognitive tests, including the open field test, Y maze, and novel object recognition test. Our results were following the results of previous studies. For example, Li et al. demonstrated that the combined treatment of Bone marrow-derived Neural Stem Cells (NSCs) with fasudil in an experimental Parkinson's disease (PD) mouse model further improves motor capacity by demonstrating more potent anti-inflammatory and antioxidant effects (Li et al., 2017). Fasudil may induce these effects by increasing motor neuron survival, inhibiting axonal degeneration, enhancing axonal regeneration, and modulating microglial function, leading to the prolongation of survival and improvement of motor function as also seen in experimental models of Amyotrophic Lateral Sclerosis (ALS) (Koch et al., 2020, Takata et al., 2013). Furthermore, fasudil could ameliorate the cognitive symptoms by reinstating PI3-kinase mediated upregulation of oxidative stress biomarker (eNOS) and control over brain proinflammatory factor regulator (NFκB activity) in a rat model of AD (Kumar and Bansal, 2018) and reducing neuronal apoptosis (Wei et al., 2021). Additionally, evidence about fasudil's effect on improving novel object recognition deficits in MK-801-treated mice (Takase et al., 2022) and rescue working memory deficits and object recognition tasks in an Oligophrenin-1 mouse model of intellectual disability (Meziane et al., 2016) also exists. Moreover, Fasudil could also attenuate delayed neuropsychologic sequelae by inhibiting inflammation and oxidative stress and downregulating the Rho/ROCK pathway in the Delayed Neuropsychologic Sequelae (DNS)mice model (Yan et al., 2021). Abnormal and persistent neuroinflammation, along with heightened oxidative stress, can result in impaired neuronal function and cell death. Therefore, neuroinflammation and oxidative stress are linked pathological features in many brain disorders, such as neurodegenerative and neuropsychiatric diseases (Zafar, 2023), and contribute to functional impairments. Consequently, addressing these issues may have therapeutic effects, as observed in our study. Furthermore, they were discussed in the following sections.

MDA is a marker of oxidative stress, while SOD is an antioxidant enzyme that helps neutralize harmful free radicals (Zhang et al., 2015). High MDA levels and low SOD activity are associated with oxidative stress, which can lead to cognitive impairment and neuroinflammation (Keyhanifard et al., 2023, Bejeshk et al., 2023). Therefore, a decrease in MDA levels and an increase in SOD activity, as seen with specific interventions, can contribute to improved cognitive function and motor skills (Keyhanifard et al., 2023, Bejeshk et al., 2023, Bacanoiu et al., 2023). Fasudil has been found to decrease MDA content and promote SOD activity, indicating its potential to activate antioxidant responses (Guan et al., 2018, Ji et al., 2020). In this regard, our study also demonstrated the improving effect of Fasudil on the increasing level of MDA following LPS-induced systemic inflammation. Still, it had no significant effect on decreasing SOD after inducing the model. However, a trend to increase in its level was also seen.

The elevated levels of the pro-inflammatory cytokine TNF-alpha have been associated with neuroinflammation, neuronal loss, and cognitive decline, particularly in conditions like Alzheimer's disease (Bejeshk et al., 2023, Zahedipour et al., 2022). Conversely, the anti-inflammatory cytokine IL-10 can help regulate the immune response. Previous studies have shown that Fasudil can suppress the production of pro-inflammatory cytokines IL-1β and TNF-α in the brain [40, 43These interventions reducing TNF-alpha levels and increasing IL-10 levels may help alleviate neuroinflammation and preserve behavioral functions such as cognitive performance (Bacanoiu et al., 2023). However, our study demonstrated that Fasudil can significantly decrease the elevated levels of IL-10 and TNF-alpha, contrary to previous studies. It also shows a reduction in TNF-alpha following systemic inflammation, which led to higher levels of both cytokines. Nevertheless, our findings showed that injection of LPS caused an unexpected increase in IL-10. Several studies have examined how administering LPS increases IL-10 expression, resulting in tolerogenic effects using various models (Rocha-Gomes et al., 2022, Hobbs et al., 2018, Liu et al., 2017, Vergadi et al., 2018). Given the heterogeneity of brain regions, one explanation for the increased IL-10 is that the number of microglia in each region may vary. Based on studies correlating improvements in cognitive function with an increase in IL-10 (Barroeta-Espar et al., 2019, Tegeler et al., 2016, Vargas-Alarcón et al., 2016) and our analysis of IL-10 levels in hippocampus tissue, as well as the large number of microglia in the hippocampus area, the change in the number of microglia cells in this region may justify the increase in IL-10 with LPS administration. Our recent study showed that administering fasudil could reduce the amount of IL-10 in the LPS+fasudil group until it reached levels similar to the sham group. While further investigation is required, it appears that fasudil's neuroprotective effect in LPS-induced inflammation is accompanied by an inhibition of microglial activation in the hippocampus region (Jung et al., 2023). In summary, the reduction of oxidative stress markers such as MDA, the nonsignificant enhancement of antioxidant activity such as SOD, and the modulation of pro- and anti-inflammatory cytokines like TNF-alpha and IL-10 collectively contribute to the improvement of motor and cognitive function by mitigating neuroinflammation and oxidative stress in our study. Excess reactive oxygen species (ROS) can cause oxidative damage that results in tissue injury, prompting an inflammatory response. This response features vasodilation and heightened vascular permeability, leading to edema—swelling caused by fluid buildup in the tissues (Jazaeri et al., 2023). The glymphatic system clears ROS and ROS-related inflammation in neurodegenerative diseases (Liu et al., 2021). Recent studies have shown that the glymphatic system removes proinflammatory cytokines and chemokines, such as TNF-alpha and interleukin-1β (IL-1β) (Hsu et al., 2021). Impairment of the glymphatic system function can result in the accumulation of ROS, causing oxidative stress and cellular injury, thereby contributing to inflammation and various brain diseases (Gu et al., 2022). ROS can trigger inflammasomes in microglia, leading to the further production of inflammatory cytokines such as TNF-α and IL-1β (Kepp et al., 2010, Rainville and Hodes, 2019) and functional impairments. On the other hand, the normal function of the glymphatic system depends on the polarized distribution of AQP-4 (Rasmussen et al., 2018). Dysfunction of AQP-4 can induce ROS accumulation proinflammatory signaling, damage vital macromolecules, and induce cellular apoptosis (Roomruangwong et al., 2017). Furthermore, the AQP-4 protein plays a role in the inflammatory process by releasing proinflammatory substances like TNFα and interleukin-6 (IL-6) and participating in the neuroinflammatory process (Li et al., 2011, Cheng et al., 2012) and modulating edema. AQP4 is essential for maintaining water balance in the brain and plays a crucial role in both the formation and elimination of brain edema in pathological diseases (Tait et al., 2008). Given the significance of AQP-4 in the pathophysiology of numerous neurological disorders and its involvement in fundamental mechanisms related to high-level functions, it is reasonable to assume that AQP-4 is also implicated in various functional impairments associated with neurological disorders (Jazaeri et al., 2023).

The maintenance of water homeostasis is carried out by special water channels present in astrocytes. Optimal water homeostasis is essential for the normal functioning of neural networks (Kong et al., 2008, Li et al., 2012). The hippocampus, having the highest density of AQP-4 channels of any brain area, makes swelling astrocytes more susceptible to cognitive dysfunction (Papadopoulos and Verkman, 2013). Astrocyte inflammation may change neurotransmitter leakage by altering synaptic concentration, impairing synaptic transmission and neuronal function. Consequently, this disturbance may negatively impact learning and memory processes (Szu and Binder, 2022, Ota et al., 2013). AQP-4, by regulating dopamine levels and astrocyte function, may play an essential role in motor function. Motor neuron function and integrity depend on AQP-4 expression levels (Nesic et al., 2006, Wu et al., 2014) and, most importantly, the astrocytic environment (Zou et al., 2019), which is regulated by AQP-4 (Seifert et al., 2006). Evidence on the participation of astrocytic AQP-4 water channels in different movement disorders and their pathophysiology, such as stroke (Catalin et al., 2018) and PD (Fan et al., 2008), further supports the role of this channel in motor function. There is evidence about the AQP-4 function in cognitive abilities, documented by 1) participation in basic mechanisms affecting cognitive functions such as Long-term potentiation (LTP)and Long-Term Depression (LTD) (Scharfman and Binder, 2013, Szu and Binder, 2016), and inducing specific cognitive impairments in-vivo studies with AQP-4 knockout models (Lu et al., 2008, Skucas et al., 2011), and 2) having a role in neurological disease with cognitive impairments (Hubbard et al., 2018). The evidence demonstrates that the destructive effect of systemic inflammation, as induced by LPS, may be caused by AQP-4 dysregulation. Sheikh et al. showed that fasudil decreased AQP-4 levels in mice with focal cerebral ischemia by reducing proinflammatory mediators, including TNF-α and IL-1β (Leonardo and Fregni, 2023). It seems that by downregulating AQP-4, Fasudil could suppress oxidative stress (decreasing ROS accumulation) and finally improve different cerebrum functions by the secretion of proinflammatory factors such as TNFsafe -α.

## 5 Limitation

With increasing degrees and duration of systemic inflammation, the vascular blood–brain barrier (BBB) becomes more permeable to solutes, undergoes an increase in lymphocyte trafficking, and is infiltrated by innate immune cells; endothelial cell damage may occasionally occur (Galea, 2021), making the person more prone to cognitive problems (Barisano et al., 2022). However, there is hope in the form of fasudil, which has been found to have a protective effect on the Blood-Brain Barrier (BBB) integrity, potentially offering cognitive benefits (Sato et al., 2022). On the other hand, AQP-4 has an essential role in BBB integrity, which was not investigated in our study. It is suggested that the role of BBB in fasudil's effect on AQP-4 be investigated, offering a promising avenue for future research.

## 6 Conclusion

Our study's findings are significant in the field of neuroscience and pharmacology. Since oxidative stress and inflammation modulation are crucial in determining the functional consequences following central nervous system (CNS) insults, the down-regulation of AQP-4 induced by fasudil, as seen in our study, can improve functional impairments of systemic inflammation, probably by reducing MDA and TNF-alpha. This improvement can ultimately enhance motor and cognitive functions, which must be further investigated in future studies, furthering our understanding of CNS disorders.

## Funding

This work was supported by a grant from the Electrophysiology Research Center of Neuroscience Institute of Tehran University of Medical Sciences.

## CRediT authorship contribution statement

**Sahra Jalalkamali:** Writing – original draft, Visualization, Formal analysis, Data curation, Conceptualization. **Mohsen Ghahremani:** Visualization, Methodology, Data curation. **Vida Jashn:** Validation, Data curation. **Negin Sadat Lajevardi:** Validation, Data curation. **Seyede Zohreh Jazaeri:** Writing – review & editing, Writing – original draft. **Javad Fahanik-babaei:** Writing – review & editing, Writing – original draft, Validation, Methodology, Investigation, Formal analysis, Data curation. **Sevda Mahdipoor Koloor:** Validation, Data curation.

## Declarations of Interest

Ethics Approval All experiments were conducted according to the Guide for Care and Use of Laboratory Animals (National Institutes of Health Publication No. 80–23, revised 1996). Additionally, the Research and Ethics Committee of Tehran University of Medical Sciences ((IR TUMS.NI) reviewed and confirmed all procedures. REC. REC.1400,05.
